# Notes

**Published:** 1899-09-30

**Authors:** 


### NOTES.

The blind appear determined to show that their affliction is not always accompanied by disability, and for this purpose some hundreds of blind brushmakers recently held a demonstration in Trafalgar Square to protest against the action of the Association for the Welfare of the Blind in locking out some of their employés who struck for better pay and conditions of labour. The meeting was of course unanimous in desiring better terms, and if their work is to be regarded from a commercial point of view, and the profit accruing warrants it, naturally it is to be expected that an association bearing a title claiming goodwill towards the blind will do full justice to the claimants. If, on the other hand, the work of the blind does not pay well enough to provide a living wage for the workers, an effective appeal to the public would seem a more reasonable remedy for the evil than a strike, which, under such circumstances, is absurd.

---

If the invention shown by Mr. W. A. Wallace at Members' Mansions bears out its promise, the housewives of England will rise up and call him blessed. He claims to have made an absolutely smokeless coal. In the fires he showed, both in ordinary grates and in chafers placed in the middle of the room, no smoke arose even when fresh fuel was put on. The fire resembled a red coke fire, but with long white and blue flames arising from it. The new coal is composed of about 93 per cent. of coal dust, which has hitherto been regarded as almost waste, with about 7 per cent. of pyroligneous tar, obtained by the distillation of wood or peat in retorts, and a small quantity of caustic lime. Mr. Wallace says that in the process of mixing the ingredients a considerable quantity of oxygen becomes imprisoned in the coal, and this aids combustion. The components are well mixed together and pressed into moulds, where they are allowed to harden. Once formed into shape, the fuel does not disintegrate in burning. For domestic purposes it is moulded into circular cakes which run about 430 to the cwt. For generating steam it is sold in large perforated briquettes weighing about 10 lb. each. It is claimed that one pound of the new fuel will evaporate 14 lb. of water, while the same quantity of Welsh coal will evaporate only 12 lb. The inventor reckons that the fuel could be sold retail in London at a guinea a ton, a price which many people would not grudge if it bears out its promise. If it be true that the heat given out by the smokeless coal is greater than that of ordinary coal it might prove much more economical in the long run; but even if it were no cheaper than the ordinary smoky article what a boon the absence of the smoke would be!

